# Supplementary material for: Study of Dandelion (Taraxacum mongolicum Hand.-Mazz.) Salt Response and Caffeic Acid Metabolism under Saline Stress by Transcriptome Analysis
Source: Genes (Basel). 2024 Feb 9;15(2):220. doi: 10.3390/genes15020220 (PMC10888437; doi:10.3390/genes15020220)
Supplement: Supplementary file 1 [file genes-15-00220-s001.zip › Supplementary Table S1.pdf]

Table S1 Primers pairs for qRT-PCR

Gene ID	Function annotation	Primers(5'-3')
TbA05G077650	4-coumarate--CoA ligase	F: TCCAAACGAGACCACAATC
		R: CATAGGAGGCAAACAAGACC
TbA07G095410	Shikimate O-hydroxycinnamoyltransferase	F: GATGTGCGTGAACTTGCTT
		R: CCAAGGATGTGAGACCAACT
TbA07G106840	4-coumarate--CoA ligase	F: AGTTTGTCTTCTCGTTCCTTG
		R: ATGTGCCGATTGAGTGATT
TbA08G014310	Caffeoylshikimate esterase	F: TACCATCCAAGCTGCATTTA
		R: ATCTCTGATTGCCTTTCCC
TbA04G072330	Caffeic acid 3-O-methyltransferase	F: GTGGATACTGCACGATTGG
		R: GCTTTGTCGGAAGAGTTTGT
TbA07G012370	Caffeic acid 3-O-methyltransferase	F: GATGAAGTTGGTGGTTAAAGAAA
		R: GGAAATGCCTCAAGAAGAATC
Novel.13839	Caffeic acid 3-O-methyltransferase	F: TGGCTTTCTTGTCTTTGAGTT
		R: CTCTTGTTTGTCGTAATGGCT
novel.13840	Caffeic acid 3-O-methyltransferase	F: CTCTTGTTTGTCGTAATGGCT:
		R: TGGCTTTCTTGTCTTTGAGTT
GAPDH	Internal reference gene	F: AGTTGGTTTCGTGGTATGAC
		R: ACATGTCAGTGAACAGGTAGAC
