# Supplementary figures and images for: Study of Dandelion (Taraxacum mongolicum Hand.-Mazz.) Salt Response and Caffeic Acid Metabolism under Saline Stress by Transcriptome Analysis
Source: Genes (Basel). 2024 Feb 9;15(2):220. doi: 10.3390/genes15020220 (PMC10888437; doi:10.3390/genes15020220)

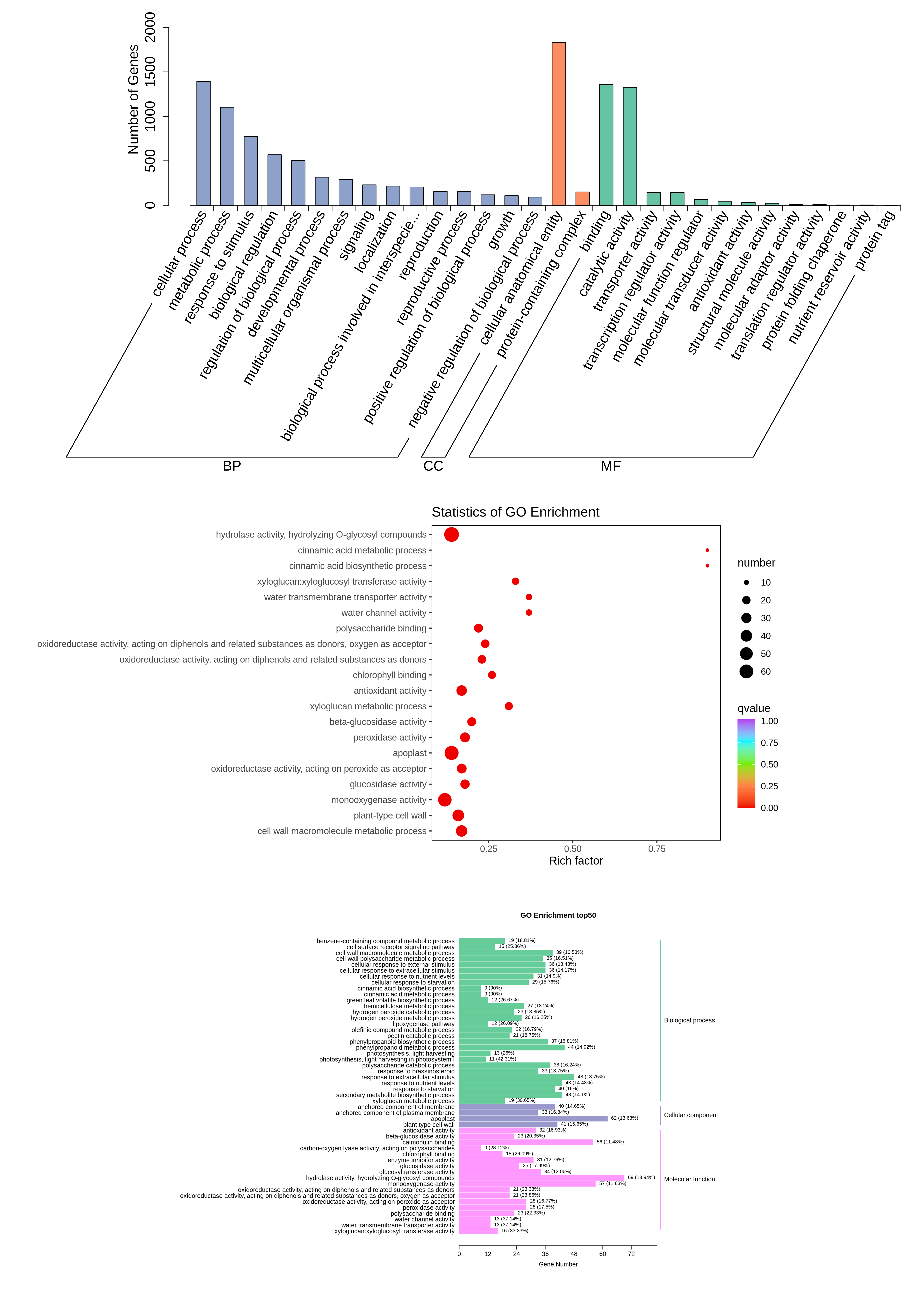

Supplement: Supplementary file 1 [file genes-15-00220-s001.zip › Supplementary Figure S1.png]
